# Selections from Foreign Journals

**Published:** 1889-07

**Authors:** 


### SELECTIONS FROM FOREIGN JOURNALS.

CUTANEOUS HORNS.—At a meeting of the Pathological Society of London Mr. Bland Sutton exhibited specimens of Cutaneous Horns growing from a sebaceous cyst in a mouse, a Papillary Horn growing from the leg of an oyster-catcher, a Horn growing from the scar of a burn on the thigh of an old woman, and Horny Material from a dermoid cyst. He then attempted to show that in many cases, especially when the horn was large and had been growing for some time, it was difficult, if not impossible, to decide whether a cutaneous horn had its origin in a wart, sebaceous cyst, or in the cicatrix of a burn. In many cases, however, the history and characters of the horn enabled us to clearly decide as to the mode of origin. In one of the mice shown to the society the base of the horn was occupied by a sebaceous cyst; in the case of the bird, the oyster-catcher, the horn had developed from a wart; whilst the third specimen, that from a woman's thigh, originated in the scar of a burn. Some competent pathologists were of opinion that, when horns grew from sebaceous cysts, the horny material exhibited a transversely animated appearance resembling a pie-crust, whilst papillary horns had a longitudinal striation. Such tests were fallacions. Sabaceous horns might be longitudinally striated, as in the mouse; whilst in the horn growing from a cicatrix the pie-crust arrangement was strikingly shown. Thus opinions formed on these grounds were very likely to lead to error. It was very propable that many horns growing from sebaceous cysts really arose from warts springing from the walls of such cysts. In order to show the difficulty of distinguishing a sebaceous from a papillary horn, two mice were shown: one had a sebaceous horn growing from its back: the other, a specimen from the museum of the Royal College of Surgeons, had a papillary horn growing from its head. In external characters the two horns were identical. In the mouse with the horn on its back, the horn rested upon a large sebaceous cyst, whilst in the second mouse the microscope showed that the horny matter had arisen from a wart. Mr. Roger Williams maintained that a sebaceous cyst was not a retention of normal secretion, but that the cells instead of undergoing the usual granulo-fatty change, became cornified, and thus were retained. An exaggeration of this process would produce a horn.—*The Lancet.*

---

Epithelioma in the Horse.—Dr. Glasgow Patterson states that epithelioma was an exceedingly rare disease among the domesticated animals, while among wild animals either living in confinement or in their natural state it was almost unknown. Mr. Bland Sutton had met with only two examples. One was in a Japanese wolf; the growth occupied the mucous membrane between the gums and the tongue. The second example occurred in the anus of a dog. Mr. M'Fadyean, demonstrator of Pathology in the Royal Veterinary College, Edinburgh, had not met with a case, and both he and Mr. Sutton agreed in the observation that tumors from the penis of the horse were generally fibromatous or papillomatous. Dr. Patteson showed specimens he had obtained from a horse ten years old. The tumor was situated on the penis, and originated in a part of the epithelium of which normally contained pigment, yet in no portion of the neoplasm could pigmented cells be found. Professor Bennett said that it had been long familiar to pathologists that carcinoma, in the form of scirrhus occurred in the lower animals. These cases were important as tending to dispose an absurd theory that cancer in man was a descendant of syphilis.—*The Lancet.*

